# Draft Genome Sequence of a Streptococcus suis Isolate from a Case of Cattle Meningitis

**DOI:** 10.1128/MRA.00153-20

**Published:** 2020-05-07

**Authors:** Ogi Okwumabua, Charles H. D. Williamson, Talima R. Pearson, Jason W. Sahl

**Affiliations:** a Department of Pathology and Population Medicine, College of Veterinary Medicine, Midwestern University, Glendale, Arizona, USA; b Department of Pathobiological Sciences, School of Veterinary Medicine, University of Wisconsin, Madison, Wisconsin, USA; c Pathogen and Microbiome Institute, Northern Arizona University, Flagstaff, Arizona, USA; University of Arizona

## Abstract

Streptococcus suis is primarily a pig pathogen and a zoonotic agent. Recently, the isolation of S. suis strain 10-36905 from a case of meningitis in cattle was reported. The draft genome sequence of this isolate demonstrates its divergent relationship with other S. suis strains.

## ANNOUNCEMENT

Streptococcus suis is a Gram-positive bacterium that primarily causes diseases in swine, such as meningitis, endocarditis, septicemia, and arthritis, and sudden death (1). S. suis is also a zoonotic agent. Human infections are often due to occupational exposure to pigs or consumption of undercooked pork (2, 3). Isolation of S. suis from dogs, cats, ruminants, and horses has been reported (1, 4–6), but whole-genome data are limited, hindering understanding of its taxonomy, biology, evolution, and host adaptability. Recently, S. suis strain 10-36905 was isolated from the brain of a calf (cattle) with meningitis that subsequently died in Wisconsin (7). In this study, we announce a draft genome assembly of 10-36905.

Genomic DNA was extracted after culture (8) and sequenced at the University of Wisconsin Biotechnology Center using a MiSeq sequencer and a MiSeq 500-bp (v2) sequencing cartridge, with paired read lengths of 250 bp after library preparation using the TruSeq Nano DNA low-throughput (LT) library prep kit (Illumina). Images were analyzed using the standard Illumina pipeline (v1.8.2). Default parameters were used for all software unless otherwise specified.

Reads were processed with Skewer (-k, 15; -l, 25) (v0.1.126) (9), and short reads (<250 nucleotides [nt]) were removed with BBTools (reformat.sh; min length, 250) (v38.61b; https://sourceforge.net/projects/bbmap/). The genome sequence was assembled from 692,810 read pairs with SPAdes (–careful –cov-cutoff auto) (v3.10.1) (10) and annotated with PGAP (11). The final genome assembly was 2,148,541 bp, distributed in 36 contigs (*N*_50_, 119,199 bp), with an average G+C content of 39.78%. Genome analysis revealed 2,049 coding genes. The isolate was assigned a new multilocus sequence type for S. suis (12), namely, sequence type 1289 (ST1289) (https://pubmlst.org/ssuis/) (13).

**FIG 1:**
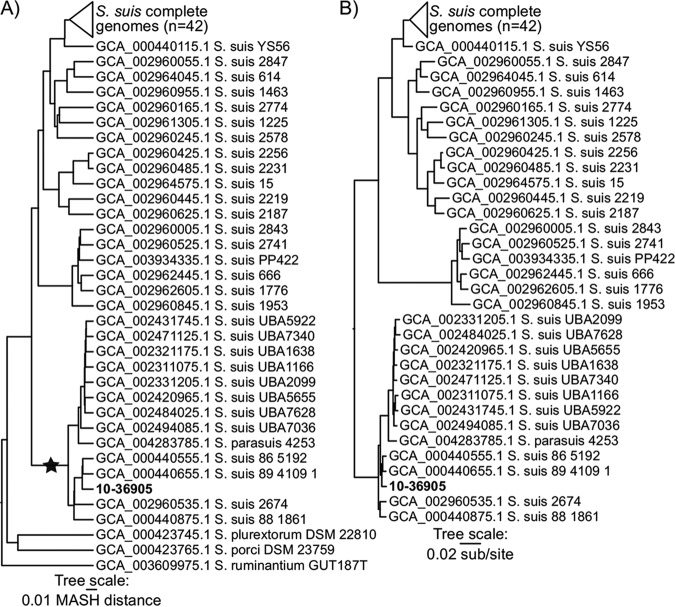
Clustering of *Streptococcus* genomes based on Mash distances (A) and a concatenated core protein phylogeny of S. suis and *S. parasuis* genomes (B). The newly sequenced genome is in bold type. The clade labeled with a star in panel A includes divergent S. suis genomes to which 10-36905 was compared with Mash; the average MASH distance between 10-36905 and other genomes within this clade is 0.042.

A comparison of the genome assembly of 10-36905 to publicly available *Streptococcus* genomes (*n* = 622) with Mash (k = 21, s = 1,000,000) (v2.2) (14) distances followed by clustering with the UPGMA within QIIME (v1.9.1) (15) (subset of 77 genomes; Fig. 1A) showed that it clustered with S. suis. Proteins common to a set of 73 S. suis and 1 S. parasuis genomes were identified with the LS-BSR tool (16) (v1.0.3) (TBLASTN [17] alignment option), extracted from genome assemblies with TBLASTN, and aligned with MUSCLE (v3.8.31) (18) using the extract_core_genome.py tool within LS-BSR. A maximum likelihood phylogeny was generated with IQ-TREE (v1.6.10) (-m MFP) (19) on the alignment of 185,775 amino acids using the best-fit model identified by Modelfinder (20) (Fig. 1B). Results demonstrate that the isolate is most closely related to other S. suis genomes but falls outside the large clade of complete S. suis genomes (average Mash distance, 0.107; *n* = 42) and with other more divergent S. suis genomes (average Mash distance, 0.042; *n* = 13), including a recently sequenced *S. parasuis* genome (ENA accession no. GCA_004283785.1). The previously identified extracellular protein factor, muramidase-released protein, and suilysin (21–23) in swine S. suis were not identified in this strain. The availability of this assembly opens possibilities for genetic studies of S. suis of cattle origin, particularly pathogenicity analysis, molecular evolution, host adaptability, and therapeutic and vaccine development.

### Data availability.

Data are available from NCBI under BioProject PRJNA590796. The whole-genome shotgun project was deposited at DDBJ/ENA/GenBank under accession no. WNXH00000000. The version described here is the first version, WNXH01000000.
